# Anti HSV-1 Activity of Halistanol Sulfate and Halistanol Sulfate C Isolated from Brazilian Marine Sponge *Petromica citrina* (Demospongiae)

**DOI:** 10.3390/md11114176

**Published:** 2013-10-29

**Authors:** Tatiana da Rosa Guimarães, Carlos Guillermo Quiroz, Caroline Rigotto, Simone Quintana de Oliveira, Maria Tereza Rojo de Almeida, Éverson Miguel Bianco, Maria Izabel Goulart Moritz, João Luís Carraro, Jorge Alejandro Palermo, Gabriela Cabrera, Eloir Paulo Schenkel, Flávio Henrique Reginatto, Cláudia Maria Oliveira Simões

**Affiliations:** 1 Laboratory of Natural Products, Department of Pharmaceutical Sciences, Universidade Federal de Santa Catarina, Florianópolis 88040-900, SC, Brazil; E-Mails: tatyguimaraes@gmail.com (T.R.G.); simonequintana@hotmail.com (S.Q.O.); terezarojo@gmail.com (M.T.R.A.); ebianco@chemist.com (E.M.B.); mizabelgm@gmail.com (M.I.G.M.); eloirschenkel@gmail.com (E.P.S.); freginatto@hotmail.com (F.H.R.); 2 Laboratory of Applied Virology, Department of Microbiology, Immunology and Parasitology, Universidade Federal de Santa Catarina, Florianópolis 88040-900, SC, Brazil; E-Mails: carlosguillermo.quiroz@gmail.com (C.G.Q.); rigottocarol@gmail.com (C.R.B.); 3 Laboratory of Porifera, National Museum, Universidade Federal do Rio de Janeiro, Rio de Janeiro 20940-040, RJ, Brazil; E-Mail: joao.porifera@gmail.com; 4 UMYMFOR—Department of Organic Chemistry, FCEN—University of Buenos Aires, Buenos Aires C1428EGA, Argentina; E-Mails: palermo@qo.fcen.uba.ar (J.A.P.); gabyc@qo.fcen.uba.ar (G.C.)

**Keywords:** antiviral activity, HSV-1, marine sponge, *Petromica citrina*, sulfate sterols

## Abstract

The *n*-butanol fraction (BF) obtained from the crude extract of the marine sponge *Petromica citrina*, the halistanol-enriched fraction (TSH fraction), and the isolated compounds halistanol sulfate (**1**) and halistanol sulfate C (**2**), were evaluated for their inhibitory effects on the replication of the Herpes Simplex Virus type 1 (HSV-1, KOS strain) by the viral plaque number reduction assay. The TSH fraction was the most effective against HSV-1 replication (SI = 15.33), whereas compounds **1** (SI = 2.46) and **2** (SI = 1.95) were less active. The most active fraction and these compounds were also assayed to determine the viral multiplication step(s) upon which they act as well as their potential synergistic effects. The anti-HSV-1 activity detected was mediated by the inhibition of virus attachment and by the penetration into Vero cells, the virucidal effect on virus particles, and by the impairment in levels of ICP27 and gD proteins of HSV-1. In summary, these results suggest that the anti-HSV-1 activity of TSH fraction detected is possibly related to the synergic effects of compounds **1** and **2**.

## 1. Introduction

The drug of choice for the prophylaxis and treatment of Herpex Simplex Virus (HSV) infections is acyclovir (ACV), which selectively inhibits HSV DNA replication with low host-cell toxicity. However, the intensive use of antiviral drugs has led to the emergence of resistant viruses [1,2,3]. Recently, De Clercq [4] described the evolution of antiviral agents against some viral infections, including HSV, confirming that the search for new antiviral agents is still relevant.

Pharmaceutical interest in marine organisms has provided thousands of new and novel compounds that have shown important biological properties, such as anticancer, antiviral, antiprotozoal, and antibacterial activities [2,5,6,7,8]. In this context, marine sponges have been a prolific source of diverse secondary metabolites with complex and unique structures [2,9,10,11,12,13]. Some of them were used as lead compounds to obtain new drugs that are currently used in clinics, such as acyclovir, vidarabine, cytarabine, eribulin mesylate, and others, that are now in clinical stages of evaluation such hemiasterlin [14,15,16]. In addition, several highly active compounds from marine sponges have been reported as new biologically active structures [17,18,19,20,21,22,23,24].

*Petromica citrina* (Porifera, Demospongie) belongs to a marine sponge genus that occurs only on the Brazilian coast [25]. There are few studies with this species, and most of them describe the evaluation of different pharmacological properties such as antibacterial and antiviral activities for its aqueous extracts [26,27] and *n*-butanol fraction [28]. Moreover, a restricted number of chemical investigations and a few bioactive constituents have been reported, in particular, a sulfated steroidal compound, identified as halistanol sulfate [29,30].

Recently, our research group described the anti-herpes activity of the *n*-butanol fraction of *P. citrina* [28]. Thus, the aim of this investigation was to determine, through a bioguided study, the active compounds responsible for the anti-HSV-1 activity detected.

## 2. Results and Discussion

### 2.1. Bioguided Fractionation of the *n*-Butanol Fraction of *P. citrina*

In a previous screening of the anti-infective potential of marine invertebrates and seaweeds [28], we observed a promising activity for the *n*-butanol fraction (BF) obtained from the ethanolic crude extract of this sponge that led us to perform this study. Our goal was to isolate, through a bioguided study, the anti-herpes bioactive metabolites present in this fraction.

**Figure 1:**
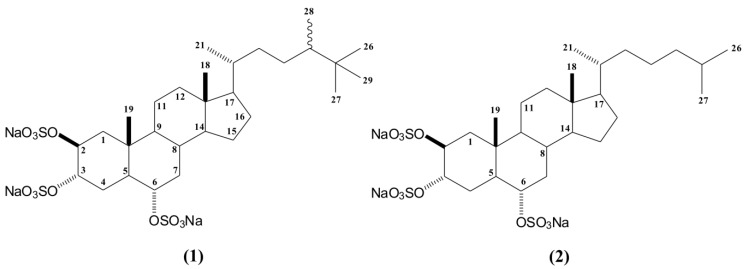
Structures of halistanol sulfate (**1**) and halistanol sulfate C (**2**).

First, the BF fraction was submitted to several Sephadex LH-20 chromatography procedures yielding five fractions (Sep-1 to Sep-5), which were pooled based on thin-layer chromatography (TLC) similarity. Among these fractions, only fraction Sep-5 showed anti HSV-1 activity and was submitted to NMR analysis. The ^1^H NMR spectrum of Sep-5 displayed characteristic signals of the presence of halistanol sulfates as the major compounds. These major compounds were isolated by C18 column chromatography, yielding compounds **1** and **2** (Figure 1).

The complete structure of compound **1** was determined based on HSQC, HMBC, and COSY spectra, as well as by ESI mass spectrometry and by comparison with literature data [29,30,31,32,33]. The presence of three sulfate groups in the structure could be clearly defined by ESI mass spectrometry (*m*/*z* 731 [M − Na]^−^, *m*/*z* 611 [M − NaHSO_4_], *m*/*z* 491 [M − (NaHSO_4_)_2_] and *m*/*z* 354 [M − (NaHSO_4_)_3_]). These sulfate groups was also supported by the IR band (1230 cm^−1^).

In addition, the ^1^H NMR spectrum of compound **1** showed carbinol signals at δ_H_ 4.83 (sl), δ_H_ 4.76 (sl; *J* = 1.8 Hz), and δ_H_ 4.20 (dt; *J* = 11.0; 4.4 Hz), corresponding in the HSQC spectrum to the signals at δ_c_ 75.6 (CH-2 and CH-3), and δ_c_ 78.8 (CH-6), respectively. These data, together with characteristic signals of two methyl singlets at δ 0.70 (CH_3_-18) and δ 1.07 (CH_3_-19), suggested a sulfated sterol nucleus. The structure of the side chain of compound **1** was elucidated by analysis of 2D NMR data. The NMR spectra showed the presence of a side chain containing two secondary methyls at δ 0.95 (d; *J* = 6.4 Hz) and δ 0.84 (d; *J* = 6.8 Hz) attributed to positions C_21_ and C_28_, also based on HMBC data. The ^1^H NMR spectra revealed a singlet at δ 0.86 (9H), which was connected to carbon at δ 27.9, suggesting a *t*-butyl group on the side chain. HMBC correlations of carbons at δ 27.9 (C_26_, C_27_ and C_29_), δ 34.2 (C_25_), and δ 45.5 (C_24_) to the proton at δ 0.86 confirmed that C_26_, C_27_, and C_29_ were connected to C_25_. Therefore, compound **1** was identified as halistanol sulfate, a steroid previously reported for marine sponges such as *Halichondria* cf. [31], *Epipolasis* sp. [32], *Petromica ciocalyptoides* [29], *Haliclona* sp. [33], and *Petromica citrina* [30].

Halistanol sulfate (HS) was first reported in 1981 by Fusetani *et al.* [31] and, in that work, the authors only showed the ^13^C NMR data of HS. New compounds of the halistanol sulfate series (halistanol sulfates A to H) were isolated in the subsequent years [32,34], but the nomenclature and the chemical shift values in the ^1^H NMR spectra of the side chain are still not completely defined [29,32]. Therefore, it is important that the details of the structural elucidation of compounds **1** and **2** are also presented.

Compound **2** also showed the same halistanol steroidal nucleus signals, but with a shorter side chain, which was inferred by NMR data together with the information of the ESI mass spectrum. Moreover, the ESI/MS spectrum showed the presence of three sulfate groups (*m*/*z* 703 [M − Na]^−^; *m*/*z* 583[M − NaHSO_4_]; *m*/*z* 463 [M − (NaHSO_4_)_2_] and *m*/*z* 340 [M − (NaHSO_4_)_3_]) in the structure. As well as for compound **1** the presence of sulfate groups in the structure was also supported by the IR band (1226 cm^−1^). Although the ^1^H-NMR spectra of compound **1** displayed two methyl doublets at δ 0.95 and δ 0.84 on the side chain, corresponding to C_21_ and C_28_, respectively, the ^1^H NMR of compound **2** only one doublet signal at δ 0.94 (d; *J* = 6.6 Hz), corresponding to the C_21_ methyl group. In addition, the ^1^H NMR data did not show a *t*-butyl group at the end side of the chain. Furthermore, two new methyl signals at δ 0.87 (d; *J* = 6.6 Hz) and δ 0.89 (d; *J* = 6.6 Hz) were identified. Considering the *J* values of these protons, we could suggest the presence of an isopropyl on the side chain. Thus, based on the data obtained, compound **2** was identified as halistanol sulfate C, a steroid previously reported for *Pseudoaxinissa digitata* [34] and *Epilopasis* sp. [32]. As far as we are aware, this is the first report of halistanol sulfate C for *Petromica citrina.*

Sulfated sterols have been described from a wide variety of marine organisms, such as sponges and echinoderms. Several of these sterols have a great structural diversity and broad spectrum of biological activities [35,36,37,38,39].

The first reported compound of the halistanol family was halistanol sulfate, isolated from the marine sponge *Halichondria* cf. moorei Bergquist [31]. Important biological activities have been reported for this steroid sulfate, such as anti-HIV effects [38], cytotoxic activity against human hepatoma cells (QGY-7701), and chronic myelogenous leukemia cells (K562) [40]. Afterwards, the same compound was isolated from *Petromica ciocalyptoides* and *Topsentia ophiraphidites*, showing inhibitory activity of *Leishmania tarentola* [29] and a wide spectrum of activity against resistant bacteria such as *Staphylococcus aureus*, *Staphylococcus epidermidis*, *Enterococcus faecalis*, *Mycobacterium fortuitum*, and *Neisseria gonorrheae* [30].

**Figure 2:**
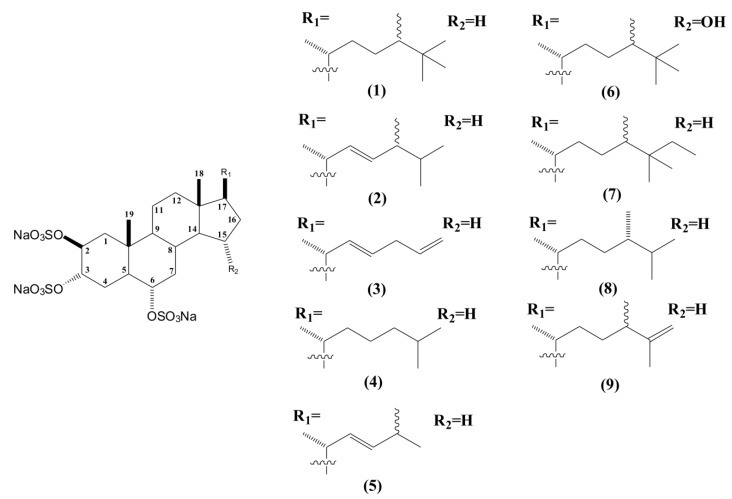
Structures of halistanol sulfate (**1**) and derivatives halistanol sulfates A to H (**2**–**9**).

Thus far, eight sulfated sterols have been described with this fundamental nucleus and named as halistanol sulfates A to H (Figure 2). All of them are characterized by the same 2β, 3α, 6α-trisulfoxy functionalities, differing only in their side chains [32,34,35]. The most promising pharmacological activities described for these compounds were the anti-HIV-1 and anti-HIV-2 effects for halistanol sulfates F and G [32].

In addition, there are many other reports about different members of the halistanol series that have shown important pharmacological properties. One of the first reported members of this series was ibisterol sulfate, isolated from *Topsentia* sp., which showed anti-HIV activity [41]. Other examples of halistanol-type compounds with antiviral activity are weinbersterol disulfates A and B isolated from the sponge *Petrosia weinbergi* which exhibited activity against leukemia virus (FeLV), mouse influenza virus (PR8), and mouse coronavirus (A59) replication [42].

As compounds with sulfated groups are described to have antiviral properties [34,38,43,44,45,46], and due to the anti-herpetic activity shown by the BF fraction, we decided to verify the anti-HSV-1 activity of compounds **1** and **2** and the TSH fraction and to elucidate their mode of action.

### 2.2. Antiviral Activity

The evaluation of potential antiviral activity of *P. citrina* fractions [Sep-1, Sep-2, Sep-3, Sep-4, and Sep-5 (TSH fraction)] as well as the isolated compounds (**1** and **2**) was performed against HSV-1 (KOS strain) using the viral plaque number reduction assay.

According to the results obtained (Table 1), the isolated compounds **1** (SI = 2.46) and **2** (SI = 1.45) showed weak activity. On the other hand, the TSH fraction that contains these compounds as the major constituents showed the most promising activity (SI = 15.33).

As it is important to understand the targets and the mode of action of a potential useful new antiviral agent, a set of experiments was carried out to determine the stages at which the most active samples (TSH fraction and compounds **1** and **2**) affect the viral replication cycle.

Pretreatment of Vero cells with TSH fraction and compounds **1** and **2** for three hours before viral infection showed that these samples did not affect viral infectivity suggesting that they did not exert protective effects against the HSV-1 infection process (data not shown).

**Table 1:** Cytotoxicity and anti-herpetic activity of samples obtained from *Petromica citrina*.

Samples	CC_50_^a^	IC_50_^b^	SI ^c^
BF fraction	>500.00	100.99 ± 19.65	>4.95
Sep-1 fraction	388.38 ± 2.54	NI	—
Sep-2 fraction	430.13 ± 10.76	NI	—
Sep-3 fraction	45.37 ± 15.17	NI	—
Sep-4 fraction	23.83 ± 11.80	NI	—
Sep-5 (TSH fraction)	44.05 ± 2.52	2.87 ± 0.78	15.33
Compound **1**	13.83 ± 3.75	5.63 ± 1.37	2.46
Compound **2**	11.89 ± 4.02	6.09 ± 1.51	1.95
ACV	>2000	3.45 ± 0.42	>580

Values represent the mean ± standard deviations of three independent experiments. NI = no inhibitory activity; ^a^ 50% cytotoxicity concentration, Vero cells (µg/mL); ^b^ 50% viral inhibitory concentration, HSV-1 (KOS strain) (µg/mL); ^c^ Selectivity index (SI = CC_50_/IC_50_).

The direct virus inactivating activity of the tested samples, in the absence of cells, was also evaluated. It was also observed that the TSH fraction and compounds **1** and **2** were able to reduce HSV-1 infectivity at concentrations 8×, 12× and 6× lower than their IC values (Table 2). This is in accordance with previous studies that have reported the virucidal activity of halistanol sulfates F and H against HIV replication [34,38].

**Table 2:** Virucidal, attachment and penetration inhibitory effects of samples obtained from *Petromica citrina* on HSV-1 (KOS strain) replication.

Samples	VC_50_^a^	AC_50_^b^	PC_50_^c^
TSH fraction	0.38 ± 0.12	6.41 ± 0.63	2.45 ± 1.33
Compound **1**	0.48 ± 0.04	7.84 ± 1.03	5.67 ± 0.47
Compound **2**	1.08 ± 0.36	12.26 ± 3.58	8.90 ± 1.86
Dextran sulfate	NA	<15.62	<15.62

Values represent the mean ± standard deviations of three independent experiments. NA = no activity; ^a^ 50% virucidal concentration (µg/mL); ^b^ 50% attachment inhibitory concentrations (µg/mL); ^c^ 50% penetration inhibitory concentrations (µg/mL).

In order to determine whether these samples were able to interfere with early events of HSV infection, their effects on HSV-1 attachment and penetration were investigated separately. All the tested samples inhibited virus attachment and penetration, as shown in Table 2. Therefore, the inactivation of HSV-1 could be related to virions binding to heparan sulfate receptors, inhibiting these two early stages of viral replication. Other natural sulfated molecules, such as sulfated polysaccharides, were also active against HIV, HSV-1, and HSV-2 replication [43,44,45,46], inhibiting these same early events of viral replication.

It is well documented that the antiviral potency of sulfated compounds depends on their degree of sulfation [4,47]. Moreover, it has become clear that the antiviral properties of sulfated compounds are not only a simple function of their detailed structural features, but also of their charge density. For instance, a highly charged molecule is more likely to interfere with electrostatic interactions between the positively charged region of a viral glycoprotein and the negatively charged HS chains of the cell-surface glycoprotein receptor, which could explain the blockade of viral attachment and penetration by competitive inhibition [48].

Additionally, we also tested the anti HSV-1 activity of halistanol disulfate (DS) and halistanol monosulfate (MS) (data not shown). It was observed that DS was less active than halistanol sulfate and halistanol sulfate C (compounds **1** and **2**, respectively; both trisulfated derivatives) as well as the MS being inactive against HSV-1.

**Figure 3:**
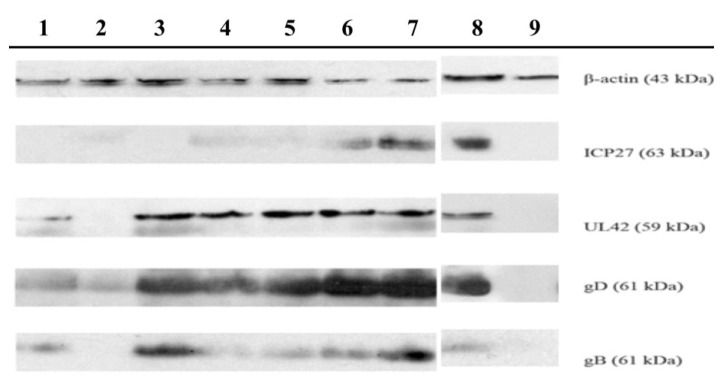
Effects of samples obtained from *Petromica citrina* on HSV-1 (KOS strain) proteins expression.

In view of the fact that our results suggest that TSH fraction and compounds **1** and **2** affect the early stages of HSV replication, we also investigated the effects of these samples on protein expression during HSV-1 replication by Western blotting (Figure 3). The results showed that the TSH fraction was the only sample that reduced the expression of all the tested proteins, in a concentration-dependent manner. Nevertheless, the (α) immediate ICP27 protein expression of HSV-1 (KOS strain) was reduced by all the tested samples, confirming that they interfere with the early events of HSV-1 replication. In addition to this event, a concentration-dependent inhibition of gB glycoprotein synthesized in the late phase (γ) of HSV-1 replication was also observed. Only TSH fraction reduced gD expression. These results suggest that an alteration in immediate early protein expression could affect the expression of late proteins. This statement is supported by the findings of Fontaine-Rodrigues and Knipe [49], who demonstrated that ICP27 is required for the efficient expression of HSV late proteins.

Given the fact that TSH fraction, compounds **1** and **2** seemed to act in a different way than ACV, the potential synergistic effects between them were tested at different concentrations (Table 3). The results obtained suggest a strong synergism between TSH fraction, compound **2** and compounds **1** + **2** and ACV, and a moderate synergism when compound **1** was tested with this drug, at the higher concentration (2 × IC_50_). When compounds **1** and **2** were tested in association, a strong synergism was also detected, at the three tested concentrations. In relation to the other combinations, a slight or a moderate antagonism was detected, exception to the association of ACV and TSH fraction, at the intermediate concentration (1 × IC_50_), when an additive effect was detected.

Treated and untreated Vero cells were analyzed by SDS-PAGE/Western blotting using specific antibodies for viral (ICP27. UL42. gD or gB) and cellular (β-actin) proteins. Column 1: Infected cells treated with 1 × CI_50_ TSH fraction; Column 2: Infected cells treated with 2 × CI_50_ TSH fraction; Column 3: Infected cells treated with 1 × CI_50_ Compound **1**; Column 4: Infected cells treated with 2 × CI_50_ Compound **1**; Column 5: Infected cells treated with 1 × CI_50_ Compound **2**; Column 6: Infected cells treated with 2 × CI_50_ Compound **2**; Column 7: Infected cells without treatment (Viral control—VC); Column8: Infected cells treated with 2 × CI_50_ acyclovir (ACV); and Column 9: Not infected cells (cellular control—CC).

**Table 3:** Synergistic effects of combination of TSH fraction and compounds **1** and **2** with acyclovir (ACV) on anti-HSV activity.

Compounds Combination Ratio	2 × IC_50_	1 × IC_50_	0.5 × IC_50_
	Experimental CI Values (Description—Graded Symbols)		
ACV + TSH fraction	0.113	1.033	1.253
	(++++)	(±)	(− −)
ACV + Compound **1**	0.745	1.21	1.243
	(++)	(− −)	(− −)
ACV + Compound **2**	0.102	1.169	1.202
	(++++)	(−)	(− −)
Compound **1** + Compound **2**	0.197	0.146	0.131
	(++++)	(++++)	(++++)
ACV + Compound **1** + Compound **2**	0.104	1.131	1.121
	(++++)	(−)	(−)

CI, combination index, a quantitative measure calculated by Calcusyn Software. This index quantifies the interaction between the tested compounds as described by Chou *et al.* [50]. In detail, CI from 0.1 to 0.3 means strong synergism (++++), 0.7 to 0.85 means moderate synergism (++), 0.9 to 1.1 means additive effect (±), 1.1 to 1.2 means slight antagonism (−), and 1.2 to 1.45 means moderate antagonism (− −). Obtained values represent the mean of three independent experiments.

The observed synergism between these samples and ACV could be explained by the fact that the samples act in different steps of HSV-1 replication than those affected by this anti-herpes drug, which could be considered an interesting result. Therefore, the most important result obtained was when compounds **1** and **2** were tested in association showing that the detected anti-HSV activity could be explained by the strong synergic effects of these major compounds present in the TSH fraction. Other natural compounds with anti-herpes activity, such as sulfated polysaccharides [44,45], docosanol [51], and oxiresveratrol [52] have already been reported to present synergistic effects with ACV, which corroborate our results.

## 3. Experimental Section

### 3.1. General Experimental Procedures

General 1D and 2D NMR experiments were performed on a Bruker Avance 2 (500 MHz) instrument at 500 MHz for ^1^H and 125 MHz for ^13^C. All spectra were recorded in CD_3_OD using the signals of residual non-deuterated solvent as internal reference. Mass spectrometric analyses were performed using a Bruker micrOTOF-Q II mass spectrometer (Bruker^®^ Daltonics, Billerica, MA, USA), equipped with ESI. Multi-point mass calibration was carried out using a mixture of sodium formate from *m*/*z* 50 to 900. Data acquisition and processing were carried out using the Bruker Compass Data Analysis version 4.0 software supplied with the instrument. All the analytical solutions (0.5 mg/mL) were prepared using methanol LCMS grade. Compounds were infused into the source using a KDS 100 syringe pump (KD Scientific, Holliston, MA, USA) at a flow rate of 180 mL/min. General MS conditions: Capillary 3.5 kV (negative ion mode), dry heater 180 °C, nebulizer 0.4 bar, dry gas (N2), 4 L/min (UMYMFOR/UBA). Silica gel 60 (70–230 mesh) Merck^®^, RP18 (Fluka^®^, Buchs, Switzerland), Sephadex LH-20 (GE healthcare^®^, Chalfont St Giles, UK), TLC analysis was performed on Silica gel F_254_ and RP18 plates (Sigma-Aldrich^®^, St. Louis, MO, USA).

### 3.2. Sponge Collection

*Petromica citrina* was collected from January to July 2010 at Xavier Island (27°36′39′′ S; 48°23′32′′ W), Santa Catarina State, Brazil, at a depth of 9–17 m, and immediately frozen. The material was identified by Dr. João Luís Carraro and voucher specimens were deposited in the Porifera collection of the *Museu de Ciências Naturais da Fundação Zoobotânica do Rio Grande do Sul*, Brazil (MCNPOR 8777, 8778, 8779, 8780, 8781).

### 3.3. Extraction and Isolation of Compounds **1** and **2**

The frozen sponge (1700 g, wet) was exhaustively extracted with ethanol for three days at room temperature. The crude ethanolic extract (CHE) was filtered, the ethanol was eliminated under reduced pressure, and the gummy residue was suspended in H_2_O before being extracted successively with ethyl acetate (EtOAc) and *n*-butanol (*n*-BuOH) (3 × 500 mL) yielding three fractions: EtOAc (EAF), *n*-BuOH (BF) and aqueous residue (AR), respectively. Next, the BF fraction (2.0 g) was subjected to Sephadex LH-20 column chromatography (790 mm × 25 mm) using methanol (MeOH) as eluent. A total of 180 tubes (20 mL) were collected and combined into five fractions (Sep-1, 650 mg; Sep-2, 450 mg; Sep-3, 350 mg; Sep-4, 250 mg; and Sep-5, 300 mg) based on Silica gel thin-layer chromatography (TLC) similarity. Because the fraction Sep-5 (named TSH fraction) showed only one spot by TLC analysis, this fraction was forwarded to ^1^HNMR analysis and proved to be a mixture of halistanol sulfate (Compound **1**) and halistanol sulfate C (Compound **2**) as the major compounds.

**Figure 4:**
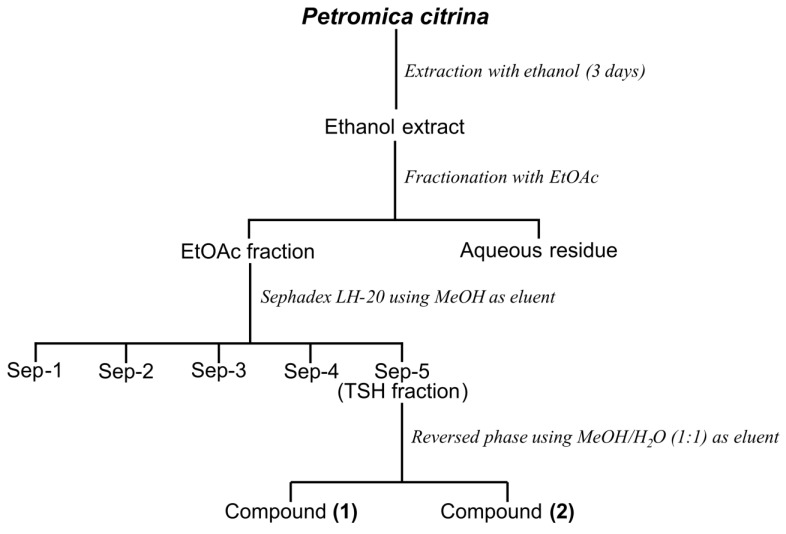
Overview of the strategy used for the purification of sulfate halistanol (compound **1**) and sulfate halistanol C (compound **2**) from the butanol fraction of *Petromica citrina*.

The TSH fraction (200 mg) was then dissolved in methanol and submitted to a reversed-phase column chromatography (300 mm × 20 mm) packed with RP18 as stationary phase and MeOH:H_2_O (1:1 v/v) as mobile phase. This procedure resulted in two isolated compounds: halistanol sulfate (30 mg, compound **1**) and halistanol sulfate C (12 mg, compound **2**). Figure 4 shows the steps of purification.

**Halistanol sulfate (1):** White amorphous powder; IR (KBr)ν_max_ 3442, 2953, 1643, 1392, 1230, 1068 cm^−1^; ^1^H NMR (CD_3_OD, 500 MHz) δ 4.83 (1H, sl, H-2), 4.76 (1H, sl, *J* = 1.8 Hz, H-3), 4.20 (1H, dt, *J* = 4.4, 10.9 Hz, H-6), 2.37 (1H, dl, *J* = 10.2 Hz, H-7a), 2.30 (1H, dl, *J* = 14.5 Hz; H-4a), 2.11 (1H, dl, *J* = 14.3 Hz, H-1a), 2.01 (1H, dl, *J* = 12.4 Hz, H-12a), 1.86 (1H, m, H-16a), 1.81 (1H, tl, *J* = 14.4 Hz, H-4b), 1.69 (1H, m, H-16b), 1.63 (1H, m, H-5), 1.64 (1H, m, H-15a), 1.58 (1H, m, H-22a), 1.55 (1H, m, H-11a), 1.54 (1H, m, H-8), 1.49 (1H, dd, *J =* 4.0, 14.3 Hz, H-1b), 1.38 (1H, m, H-20), 1.33 (1H, m, H-11b), 1.29 (1H, m, H-12b), 1.15 (1H, m, H-14), 1.12 (1H, m, H-15b), 1.10 (1H, m, H-17), 1.07 (3H, s, H-19), 1.02 (1H, m, H-7b), 0.99 (2H, m, H-24), 0.95 (3H, d, *J* = 7.4 Hz, H-21), 0.90 (1H, m, H-22b), 0.78 (2H, m, H-23), 0.86 (9H, sl, H-26, 27, 29), 0.84 (3H, d, H-28) 0.78 (1H, m, H-9), 0.70 (3H, sl, H-18); ^13^C NMR (CD_3_OD, 125 MHz): δ 78.8 (CH, C-6), 75.6 (CH, C-3), 75.6 (CH, C-2), 57.7 (CH, C-17), 57.6 (CH, C-14), 55.8 (CH, C-9), 45.5 (CH, C-24), 45.4 (CH, C-5), 43.8 (C, C-13), 41.3 (CH_2_, C-12), 40.1 (CH_2_, C-7), 39.2 (CH_2,_ C-1), 37.7 (CH_2_, C-22), 37.7 (CH, C-20), 37.7 (C, C-10), 35.2 (CH, C-8), 34.1 (C, C-25), 29.2 (CH_2_, C-16), 27.9, (CH_3_, C-26), 27.9 (CH_3_, C-27), 27.9 (CH_3_, C-29), 25.2 (CH_2_, C-15), 25.1 (CH_2_, C-4), 21.9 (CH_2_, C-11), 22.03 (CH_2_, C-23), 19.6 (CH_3_, C-21), 15.3 (CH_3_, C-19), 15.0 (CH_3_, C-28), 12.5 (CH_3_, C-18). ESI-MS *m/z* 731.2198 [M − Na]^−^ (calcd for C_29_H_49_Na_3_O_12_, 754.2100).

**Halistanol sulfate C (2):** White amorphous solid; IR (KBr)ν_max_ 3442, 2949, 1625, 1384, 1226, 1070 cm^−1^; ^1^H NMR (CD_3_OD, 500 MHz) δ 4.83(1H, m, H-2), 4.76 (1H, q, *J* = 2.7 Hz, H-3), 4H-2), 4.20 (1H, td, *J* = 11.1, 4.4 Hz, H-6), 2.37 (1H, dt, *J* = 12.3, 4.4 Hz, H-7a), 2.30 (1H, dt, *J* = 15.0, 2.8, 1.1 Hz; H-4a), 2.11 (1H, dd, *J* = 14.7, 1.7 Hz, H-1a), 2.01 (1H, dl, *J* = 12.7, 3.5 Hz, H-12a), 1.86 (1H, m, H-16a), 1.81 (1H, ddd, *J* = 15.0, 13.2, 2.8 Hz, H-4b), 1.63 (1H, m, H-5), 1.61 (1H, m, H-15a), 1.53 (1H, m, H-8), 1.52 (2H, m, H-8, H-25), 1.48 (1H, dd, *J* = 14.7, 3.9 Hz, H-1b), 1.38 (1H, m, H-20), 1.31 (1H, qd, *J* = 13.1, 3.5 Hz, H-11), 1.29 (1H, m, H-16b), 1.14 (1H, m, H-12b), 1.12 (3H, m, H-14, H-15a, H-17), 1.07 (3H, sl, H-19), 1.02 (1H, m, H-7b), 0.94 (3H, d, *J* = 6.5 Hz, H-21), 0.9–1.4 (6H, m, H2-22, H2-23, H2-24), 0.87 (3H, d, *J* = 6.6 Hz, H-26), 0.89(3H, d, *J* = 6.6 Hz, H-27), 0.78 (1H, m, H-9), 0.69 (3H, sl, H-18). ^13^C NMR (CD_3_OD, 125 MHz): δ 78.7 (CH, C-6), 75.5 (CH, C-2), 75.5 (CH, C-3), 57.6 (CH, C-17), 57.5 (CH, C-14), 55.8 (CH, C-9), 45.3 (CH, C-5), 43.8 (C, C-13), 41.2 (CH_2_, C-12), 40.8 (CH_2_, C-24), 40.0 (CH_2_, C-7), 40.0 (CH_2_, C-7), 39.2 (CH_2,_ C-1), 37.6 (C, C-10), 37.3 (CH_2_, C-22), 37.0 (CH, C-20), 35.1 (CH, C-8), 29.2 (CH_2_, C-16), 29.1 (CH, C-25), 25.1 (CH_2_, C-4), 25.0 (CH_2_, C-23), 24.9 (CH_2_, C-15), 23.1 (CH_3_, C-26), 22.9 (CH_3_, C-27), 21.8 (CH_2_, C-11), 19.1 (CH_3_, C-21), 15.2 (CH_3_, C-19), 12.5 (CH_3_, C-18). ESI-MS *m/z* 703.2032 [M − Na]^−^ (calcd for C_27_H_45_Na_3_O_12_S_3_, 726.1800).

### 3.4. Anti-HSV-1 Activity

#### 3.4.1. Virus and Cell Line

HSV-1 (KOS strain, Faculty of Pharmacy, University of Rennes, France) was propagated in Vero cells. Viral stocks were stored at −80 °C and titrated based on plaque forming units (PFU) counted by plaque assay as previously described [53].

Vero (ATCC: CCL 81) cells were grown in Eagle’s minimum essential medium (MEM; Cultilab^®^, Campinas, Brazil) supplemented with 10% fetal bovine serum (FBS; Gibco^®^, Carlsbad, CA, USA), 100 U/mL penicillin G, 100 µg/mL streptomycin, and 25 µg/mL amphotericin B (Cultilab^®^), and maintained at 37 °C in humidified 5% CO_2_.

#### 3.4.2. Cytotoxicity Assay

Vero cell viability was measured by the MTT (3-(4,5-dimethylthiazol-2-yl)-2,5-diphenyl tetrazolium bromide—Sigma-Aldrich^®^, St. Louis, MO, USA) [54]. Briefly, confluent Vero cells were exposed to different concentrations of samples for 72 h, and after incubation, the 50% cytotoxic concentration (CC_50_) of each one was calculated as the concentration that reduces cell viability by 50%, when compared to untreated controls.

#### 3.4.3. Antiviral Activity Assays

**Viral plaque number reduction assay:** To evaluate the anti-herpes activity, a plaque reduction assay was performed following the general procedures described by Silva *et al.* [55]. Vero cell monolayers were infected with approximately 100 PFU of the virus for 1 h at 37 °C, then overlaid with MEM containing 1.5% carboxymethylcellulose (CMC; Sigma-Aldrich^®^, St. Louis, MO, USA) either in the presence or absence of different concentrations of the samples. After 72 h of incubation at 37 °C, cells were fixed and stained with naphtol blue-black (Sigma-Aldrich^®^, St. Louis, MO, USA ), and the plaques were counted. The IC_50_ of each sample was calculated as the concentration that reduced the number of viral plaques in 50%, when compared to the untreated controls. ACV was used as a positive control. The selectivity index (SI = CC_50_/IC_50_) was calculated for each sample tested.

**Virucidal assay:** Mixtures of serial two-fold dilutions of samples and 4 × 10^4^ PFU of HSV-1 in serum free MEM were co-incubated for 15 min at 37 °C prior to the dilution of these mixtures to non-inhibitory concentrations (1:100) [56]. The residual infectivity was determined by the viral plaque number reduction assay, as described above.

**Pretreatment:** This assay was performed as described by Bettega *et al.* [57]. Briefly, Vero cell monolayers were pretreated with different concentrations of samples for 3 h at 37 °C prior virus infection. After washing, cells were infected with 100 PFU of HSV-1 for 1 h at 37 °C. The infected cells were washed, overlaid with MEM containing 1.5% CMC, incubated for 72 h, and treated as described earlier for the viral plaque number reduction assay.

**Simultaneous treatment:** This assay was performed as described by Silva *et al.* [55]. Briefly, 100 PFU of HSV-1 and different concentrations of samples were added concomitantly to Vero cells for 1 h at 37 °C. After washing, cells were overlaid with MEM containing 1.5% CMC, incubated for 72 h, and treated as described earlier for the viral plaque number reduction assay.

**Adsorption and penetration assays:** These assays followed the procedures described by Silva *et al.* [55], with minor modifications. Briefly, for the adsorption assay, confluent Vero cells, pre-chilled at 4 °C for 1 h, were infected with 100 PFU of HSV-1 and treated with different concentrations of samples, then incubated at 4 °C for 2 h. The unabsorbed viruses were removed by washing with cold PBS, the cells were covered with overlay medium, the temperature was raised to 37 °C, and treated as described earlier for viral plaque number reduction assay. Dextran sulfate (Sigma) was used as a positive control. For the penetration assay, 100 PFU of HSV-1 was adsorbed for 2 h at 4 °C on confluent Vero cells, after that incubated at 37 °C for 5 min to maximize virus penetration. The cells were then treated with different concentrations of samples. After 1 h at 37 °C, unpenetrated viruses were inactivated with warm citrate-buffer (pH 3.0) for 1 min. The cells were washed with PBS and treated as described above for the viral plaque number reduction assay.

**Western blotting analysis:** Procedures were performed as described by Bertol *et al.* [58]. Briefly, Vero cell monolayers were infected with HSV-1 at MOI 0.2 for 1 h. Next, residual viruses were removed with PBS and the cells were submitted to the different treatments for 18 h. The proteins were then extracted from the cells, separated on 12% SDS-polyacrylamide gel electrophoresis (SDS-PAGE), transferred to polyvinylidene difluoride (PVDF) membranes (Millipore, Billerica, MA, USA) and blocked with 5% non-fat milk in blotting buffer [25 mM Tris-HCl (pH 7.4), 150 mM NaCl, 0.1% Tween 20]. The membranes were incubated for 90 min with the following primary antibodies: Goat monoclonal antibody against ICP27 protein (1:700 dilution) (Santa Cruz Biotechnology, Santa Cruz, CA, USA); mouse monoclonal antibody against UL42 protein (1:5000 dilution) (Millipore^®^, St Charles, MO, USA); mouse monoclonal antibody against gD (1:5000 dilution) (Santa Cruz^®^ Biotechnology, Santa Cruz, CA, USA); mouse monoclonal antibody against gB (1:5000 dilution) (Millipore^®^, St Charles, MO, USA); and rabbit monoclonal antibody against beta-actin (1:5000 dilution) (Millipore^®^, St Charles, MO, USA). After washing, the membranes were incubated with the respective secondary antibodies for 1 h. The immunoblots were developed and detected using the Pierce ECL Western Blotting Substrate (Thermo^®^ Scientific, Rockford, IL, USA), according to the manufacturer’s instructions.

**Synergistic effects:** The effects of TSH fraction, compounds **1** and **2** in combination with ACV, and compounds **1** more **2** were evaluated by plaque reduction assay as described above and according to the experimental design proposed by Chou *et al.* [50]. Briefly, each sample alone or in combination was tested at a fixed ratio of its corresponding IC_50_ value (*i.e.*, at IC_50_ × 0.5 × 1 and × 2). The interaction degree between samples, based on the median-effect principle of the mass-action law, using Calcusyn software (version 2.1, Biosoft^®^, Cambridge, UK). According to the CI theorem, CI values <1, =1, and >1 indicate synergism, additive effect, and antagonism, respectively.

## 4. Conclusions

In summary, these results suggest that the TSH fraction and compounds **1** and **2** present antiherpes activity through the reduction of viral infectivity, inhibition of virus entry into the cells, and by the impairment of levels of ICP27 and gD proteins of HSV-1.

The relevant selectivity index of 15.33 of TSH fraction and its content (compounds **1** and **2**) as well as the strong synergism effects observed suggest that the detected anti-HSV activity could be explained by the synergic effects of these major compounds present in the TSH fraction.

## Abbreviations

CMC: Carboxymethylcellulose
COSY: Correlation Spectroscopy
ESI: Electrospray ionization
HMBC: Heteronuclear Multiple Bond Correlation
HSQC: Heteronuclear Single Quantum Correlation
HSV: Herpes Simplex Virus
MEM: Minimal Essential Medium
NMR: Nuclear Magnetic Resonance
PFU: Plaque Forming Units
TSH: Halistanol-Enriched Fraction
